# Advanced waveform analysis of diaphragm surface EMG allows for continuous non-invasive assessment of respiratory effort in critically ill patients at different PEEP levels

**DOI:** 10.1186/s13054-024-04978-0

**Published:** 2024-06-09

**Authors:** R. S. P. Warnaar, A. D. Cornet, A. Beishuizen, C. M. Moore, D. W. Donker, E. Oppersma

**Affiliations:** 1 Cardiovascular and Respiratory Physiology, Technical Medical Centre, University of Twente, Technohal 3184, P.O. Box 217, 7500 AE Enschede, The Netherlands; 2 Intensive Care Centre, Medisch Spectrum Twente, Enschede, The Netherlands; 3 Netherlands eScience Center, Amsterdam, The Netherlands; 4 Intensive Care Centre, University Medical Centre Utrecht, Utrecht, The Netherlands

**Keywords:** Mechanical ventilation, Respiratory failure, Neuromuscular coupling, Diaphragm, Airway occlusion pressure, Respiratory surface electromyography, Advanced signal analysis, Quality assessment

## Abstract

**Background:**

Respiratory effort should be closely monitored in mechanically ventilated ICU patients to avoid both overassistance and underassistance. Surface electromyography of the diaphragm (sEMGdi) offers a continuous and non-invasive modality to assess respiratory effort based on neuromuscular coupling (NMCdi). The sEMGdi derived electrical activity of the diaphragm (sEAdi) is prone to distortion by crosstalk from other muscles including the heart, hindering its widespread use in clinical practice. We developed an advanced analysis as well as quality criteria for sEAdi waveforms and investigated the effects of clinically relevant levels of PEEP on non-invasive NMCdi.

**Methods:**

NMCdi was derived by dividing end-expiratory occlusion pressure (Pocc) by sEAdi, based on three consecutive Pocc manoeuvres at four incremental (+ 2 cmH2O/step) PEEP levels in stable ICU patients on pressure support ventilation. Pocc and sEAdi quality was assessed by applying a novel, automated advanced signal analysis, based on tolerant and strict cut-off criteria, and excluding inadequate waveforms. The coefficient of variations (CoV) of NMCdi after basic manual and automated advanced quality assessment were evaluated, as well as the effect of an incremental PEEP trial on NMCdi.

**Results:**

593 manoeuvres were obtained from 42 PEEP trials in 17 ICU patients. Waveform exclusion was primarily based on low sEAdi signal-to-noise ratio (N_tolerant_ = 155, 37%, N_strict_ = 241, 51% waveforms excluded), irregular or abrupt cessation of Pocc (N_tolerant_ = 145, 35%, N_strict_ = 145, 31%), and high sEAdi area under the baseline (N_tolerant_ = 94, 23%, N_strict_ = 79, 17%). Strict automated assessment allowed to reduce CoV of NMCdi to 15% from 37% for basic quality assessment. As PEEP was increased, NMCdi decreased significantly by 4.9 percentage point per cmH_2_O.

**Conclusion:**

Advanced signal analysis of both Pocc and sEAdi greatly facilitates automated and well-defined identification of high-quality waveforms. In the critically ill, this approach allowed to demonstrate a dynamic NMCdi (Pocc/sEAdi) decrease upon PEEP increments, emphasising that sEAdi-based assessment of respiratory effort should be related to PEEP dependent diaphragm function. This novel, non-invasive methodology forms an important methodological foundation for more robust, continuous, and comprehensive assessment of respiratory effort at the bedside.

**Supplementary Information:**

The online version contains supplementary material available at 10.1186/s13054-024-04978-0.

## Introduction

Mechanical ventilation (MV) provides lifesaving support for patients with respiratory failure in the intensive care unit (ICU). In assisted modes of MV, work of breathing is divided between the patient and the ventilator, reducing the patient’s respiratory effort [1]. As both ventilator overassistance and underassistance may induce respiratory muscle dysfunction within hours [2], respiratory effort should be closely monitored, such that ventilatory support can adequately be tailored.

Most mechanical ventilators allow for non-invasive, yet intermittent assessment of respiratory effort based on either plateau pressure [3] or end-expiratory occlusion pressure (Pocc) [4]. The related measure of inspiratory occlusion pressure in the first 100 ms (P0.1) reflects respiratory drive rather than effort, while lacking information on respiratory mechanics and muscle function [4, 5]. Truly continuous measures of respiratory effort require invasive measurements to quantify the respiratory muscle pressure (Pmus), either directly, from oesophageal manometry (Pes), or indirectly, from invasive electromyography of the diaphragm (EAdi). As both Pes and EAdi require dedicated equipment, patient instrumentation and expertise while being highly time consuming, these techniques are not commonly applied in clinical care despite of a widely perceived need for continuous bedside monitoring.

A non-invasive, continuous, and less laborious measure of respiratory effort can be based on respiratory surface electromyography of the diaphragm (sEMGdi). To this end, Pmus is derived during assisted MV by multiplying sEMGdi activity (sEAdi) with the neuromuscular coupling index of the diaphragm (NMCdi), the latter being defined as airway pressure drop during Pocc divided by its corresponding sEAdi [6, 7]. This method has so far been confined to clinical research, as sEAdi waveforms are prone to distortion by cardiac and adjacent muscle crosstalk, while standardised signal acquisition and processing are pressingly awaited [8]. Therefore, we set out to investigate sEAdi signal quality and the effects of clinically relevant levels of PEEP on non-invasive NMCdi in mechanically ventilated ICU patients.

## Methods

### Study population

A prospective cohort was included from the mixed ICU of Medisch Spectrum Twente, a tertiary referral hospital in Enschede, the Netherlands. The protocol was approved by the medical ethical committee of Arnhem-Nijmegen, the Netherlands (CCMO-number NL75951.091.21), and registered in the Dutch Trial Register (NL9654). Written informed consent was obtained from the patients’ legal representatives. Patients were eligible if aged ≥ 18 years, invasively ventilated for at least 48 h, and ventilated in pressure support mode (SPN-CPAP/PS, Drägerwerk AG & Co. KGaA, Lübeck, Germany) with a FiO2 ≤ 60%, a SpO2 ≥ 90%, and a Richmond Agitation and Sedation Scale (RASS) score ≤ 0. Exclusion criteria were a BMI > 30 kg/m^2^ at ICU admission, a persistent pneumothorax, a history of neuromuscular disease, or pregnancy. The BMI criterion was set, as obesity adds to the complexity of acquiring adequate signal-to-noise ratios in diaphragmatic sEMG data [9].

### Data acquisition

sEMG was measured with pre-gelled Ag/AgCl electrodes (3M™ Red Dot™ 2560 electrodes, 3M Deutschland GmbH, Neuss, Germany) connected to actively shielded electrode cables (TMSi, Oldenzaal, the Netherlands). Diaphragmatic sEMG (sEMGdi) measured at the eighth intercostal space in the right anterior axillary line. An ECG lead was recorded from the sternal angle to the lower costal margin in the mid-axillary line for QRS complex detection. The skin was cleansed with alcohol before electrode application. EMG and ECG signals were acquired with a Mobi-6 device (TMSi, Oldenzaal, the Netherlands) with bipolar channels (12.2 nV/bit, amplification factor: 19.5) at a sample rate of 2048 Hz using the TMSi MATLAB interface. Airway pressure (Paw), flow (F) and volume (V) tracings from the Dräger Infinity V500 ventilator (Drägerwerk AG & Co. KgaA, Lübeck, Germany) were acquired at 100 Hz through the ventilator’s RS232 interface.

### Study protocol

Measurements were performed every other weekday, as long as the patient still met the inclusion criteria. Measurements could be called off for medical reasons at the discretion of the attending physician. An incremental PEEP trial was performed based on the clinically set PEEP, with levels according to the protocol in Table 1. Other ventilator settings were maintained as dictated by routine clinical care.

**Table 1:** Incremental PEEP trial depending on clinically used PEEP levels

	Study PEEP trial steps							
**Clinically set PEEP level**	**cmH**_**2**_**O**	**3**	**5**	**7**	**9**	**11**	**13**	**15**
	** ≤ 6**	V	V	V	V			
	**7–8**		V	V	V	V		
	**9–11**			V	V	V	V	
	**> 11**				V	V	V	V

Each PEEP step started with an adaptation phase of at least 5 min [9], extended up to 10 min in case of coughing or movement artefacts. Three spontaneous inspiratory efforts against an occluded airway (Pocc) were recorded, alternated with non-occluded breaths to resume a regular breathing pattern. Awake patients were instructed to continue quiet breathing during the end-expiratory occlusions. Additional Pocc measurements were performed in case of observable movement artefacts in the raw sEMG tracings, e.g., due to coughing or non-respiratory movements.

### Offline signal pre-processing

sEMG signals were pre-processed using the ReSurfEMG [10] library as described in more detail in Additional Files 1 and 2. The sEMG signals were bandpass filtered using a 20–500 Hz third order Butterworth filter. QRS-complexes were detected in the ECG lead and eliminated by gating with a window of 100 ms. The sEMGdi envelope, representing the electrical activity of the diaphragm (sEAdi), was calculated using a moving 200 ms RMS filter. The gating procedure was applied twice to the datasets of patients 7 and 16, because of the occurrence of two prominent ECG peaks resulting from a bundle branch block and a paced rhythm, respectively.

### Parameter calculation

Occluded breaths were automatically detected in the Paw channel as negative deflections relative to the set PEEP. The corresponding diaphragmatic activity peaks were identified from sEAdi. Respiratory muscle pressure output and neural activation during Pocc were calculated as the area under the curve relative to a moving baseline, resulting in a pressure–time-product over Pocc (PTPocc) and electrical-time-product over sEAdi (ETPdi). Moving baselines for both sEAdi and Paw were calculated by applying a moving 33rd percentile filter over a centralised window of 5 s with a step size of 200 ms as adapted from [6].

### Data analysis

sEAdi recordings showing no respiratory activity were manually excluded. If multiple inspiratory efforts occurred within one end-expiratory occlusion, only the first occluded breath was included in the analysis. All PEEP trials having at least one adequate PTPocc and ETPdi value at each PEEP level were included in the statistical analysis. To allow for between-trial comparison in the absence of a maximal voluntary manoeuvre [8], PTPocc and ETPdi values were normalised relative to their median values at a PEEP of 9 cmH_2_O, as a PEEP of 9 cmH_2_O occurred in all PEEP trials (Table 1). Normalised NMCdi was calculated from the normalised PTPocc and ETPdi values:

1 $${\text{NMC}}_{\text{di}} =\frac{{\text{PTP}}_{\text{occ},\text{ norm}}}{{\text{ETP}}_{\text{di},\text{ norm}}}$$

### Advanced data analysis

**Fig. 1:**
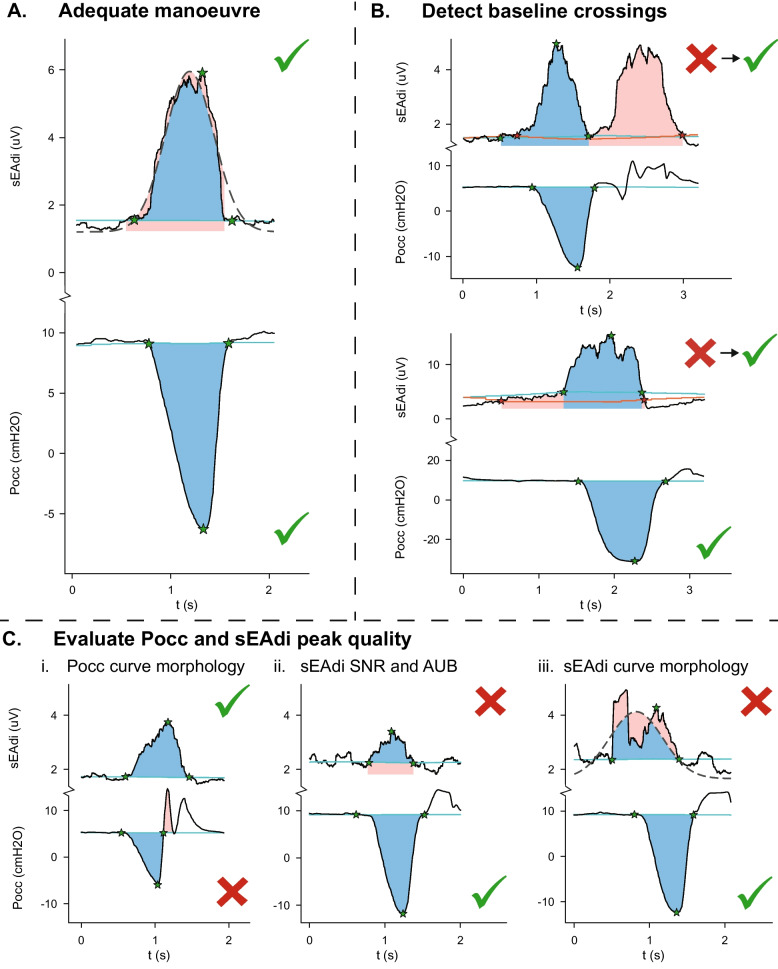
Quality criteria – A. Example of an included manoeuvre, B. Baseline crossing detection, C. Quality assessment of the i. occlusion manoeuvre (Pocc) morphology, ii. sEAdi signal-to-noise ratio (SNR) and area under the baseline (AUB), and iii. sEAdi peak morphology

Upon visual inspection, a subset of Pocc and sEAdi peaks showed physiologically improbable characteristics (Fig. 1C), introducing large uncertainty in the calculated PTPs and ETPs. Moreover, high baseline variability resulted in ill-behaved on- and offset detection in some traces (Fig. 1B). Therefore, the moving baseline and parameter calculation algorithms were improved relative to Graßhoff et al. [6] (Fig. 1B, Additional Files 1 and 2), and advanced waveform analysis was performed to assess NMCdi quality, assigning tolerant and strict criteria (Fig. 1C and Table 2). The sEAdi baseline was calculated over a 7.5 s window and amplified relative to its variance in the same window, and the PTPs and ETPs were supplemented with the area under the baseline (Fig. 1C.ii). Pocc peaks were excluded if they showed abrupt or irregular cessation of the inspiratory effort (Fig. 1C.i). sEAdi peaks were excluded if the interpeak interval of the peaks (Tdi) closely resembled the inter-beat interval of the heart (T_HR_), or if the sEAdi peaks had a substantial area under the baseline (AUB) or a low signal-to-noise ratio (SNR, Fig. 1C.ii). sEAdi peaks that differed from a bell-shape were also excluded (Fig. 1C.iii). A detailed description of these post-processing steps is provided in Additional Files 1 and 2.

**Table 2:** Quality criteria for exclusion of sEAdi peaks

		Criterium			
		Tdi (%T_HR_)	SNR (%base)	AUB (%AUC_tot_)	Bell-error (%AUC_tot_)
**Quality check**	**Manual**	N/A	N/A	N/A	N/A
	**Tolerant**	< 110%	< 140%	> 40%	> 30%
	**Strict**	< 110%	< 175%	> 30%	> 25%

*Tdi* median time between sEAdi peaks, *T*_*HR*_ median time between QRS-complexes, *SNR* Signal-to-Noise ratio, %base peak value relative to moving baseline, *AUB* area under the moving baseline curve, *AUC*_*tot*_ total area under the curve

### Statistical analysis

Data were analysed as median (interquartile range, IQR) unless stated otherwise. The effect of PEEP on NMCdi (Eq. 1) was examined using Generalised Estimating Equations (GEE) in SPSS (v. 28.0, IBM, Chicago, IL, United States) using those PEEP trials that had at least one adequate data point at each PEEP level (PTPocc and ETPdi). GEEs correct for the clustered nature of the data by estimating more robust standard errors of the regression coefficients. The effect of PEEP on NMCdi, after updating the moving baseline and applying the tolerant and strict cut-off criteria (Fig. 1C, Table 2), was assessed accordingly. P < 0.017 was considered significant, resulting from an original α of 0.05 with a Bonferroni correction for repeated testing. The effect of the exclusion criteria on repeatability and data quality was assessed according to the coefficient of variation (CoV) of NMCdi. CoV was calculated as:

2 $$\text{CoV}= \frac{\sqrt{{\text{MSE}}_{\text{w}}}}{\overline{\text{NMC}}\text{di} },$$

with MSE_w_ the mean sample variance of NMCdi expressed as the within group mean squared error, and $\overline{\text{NMC}}\text{di }$ the grand mean of NMCdi, both calculated over all included PEEP levels.

## Results

**Fig. 2:**
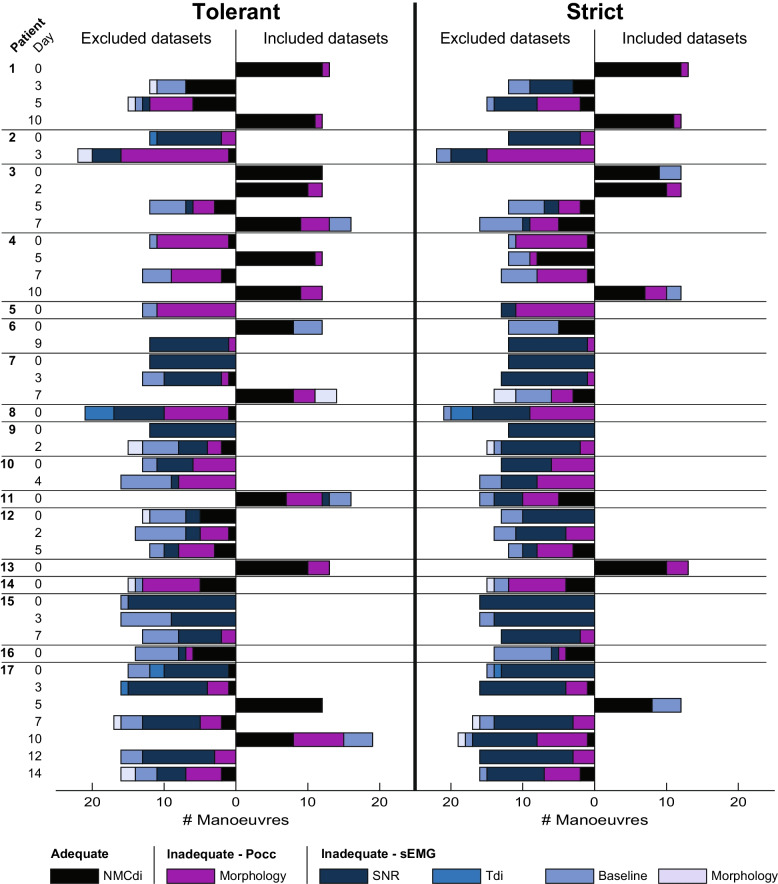
Manoeuvre permissibility per quality criterion – Permissibility of individual manoeuvres clustered by whether the PEEP trial was in- or excluded according to the tolerant (left) and strict (right) criteria. Abbreviations: NMCdi – Adequate NMCdi: both Pocc and sEAdi are adequate, Pocc – Inadequate occlusion manoeuvre morphology, SNR – Inadequately low SNR of sEAdi, Tdi – Inadequate sEAdi interpeak interval relative to the heart inter-beat interval, Baseline – Inadequately large area under the baseline, Morphology – Inadequate sEAdi due to deviation from bell-morphology

A total of 593 Pocc manoeuvres were performed over 42 PEEP trials in 17 patients (Table 3). After manual exclusion of non-respiratory waveforms, 26 PEEP trials (62%) from 13 patients were included for analysis. Application of the tolerant and strict quality criteria yielded 13 PEEP trials (31%) of 8 patients and 7 PEEP trials (17%) of 5 patients, respectively. Exclusion of individual manoeuvres was mainly based on a low SNR (N = 155, 37%), irregular or abrupt cessation of the inspiratory effort in Pocc (N = 145, 35%), and a high area under the baseline (AUB, N = 94, 23%) for the tolerant criteria (Fig. 2). For the strict criteria, manoeuvre exclusion numbers shifted towards a low SNR (N = 241, 51%), irregular or abrupt cessation of the inspiratory effort in Pocc (N = 145, 31%), and high AUB (N = 79, 17%). 70% of the low SNR exclusions occurred in patients with an above median BMI at admission (≥ 28 kg/m^2^) for the tolerant criteria, which increased slightly to 72% for the strict criteria. Regarding Pocc, 53% of the exclusions occurred in this high BMI group for both the tolerant and strict criteria, whereas exclusion fractions for a high AUB were 77% and 66% for the tolerant and strict criteria, respectively. The included PEEP trials per patient along with the quality criteria over MV duration, and the pre-measurement SpO2, RASS-score, and MV settings can be found in Additional File 3.

**Table 3:** Patient characteristics

Subject	Age (y)	Gender	BMI (kg/m^2^)	Reason of admission	Days of MV at inclusion (days)
1	55	M	25	Pneumonia—COVID19	30
2	68	F	21	Sepsis—Abdominal	2
3	68	M	30	Pneumonia—COVID19	6
4	61	M	25	Pneumonia—COVID19, PE	12
5	61	F	18	Pneumonia—COVID19	5
6	76	M	23	Pneumonia—COVID19, PE	5
7	72	M	28	Surgical—Abdominal	6
8	55	F	29	Pneumonia—Unilateral Pneumococcus, Sepsis	4
9	49	M	25	Trauma—TBI	7
10	50	M	29	Trauma—TBI	15
11	52	F	23	COPD exacerbation	7
12	75	M	28	Surgical—Cardiothoracic (Valve replacement)	17
13	75	F	23	Surgical—Cardiothoracic (Morrow)	22
14	58	M	30	Trauma—Poly	20
15	74	M	29	Surgical—AAA	8
16	61	M	28	Sepsis—Lead endocarditis	6
17	78	M	28	Pneumonia—Legionella	3

*MV* mechanical ventilation, *BMI* body mass index, *PE* pulmonary embolism, *TBI* traumatic brain injury, *COPD* chronic obstructive pulmonary disease, *AAA* abdominal aortic aneurysm

**Fig. 3:**
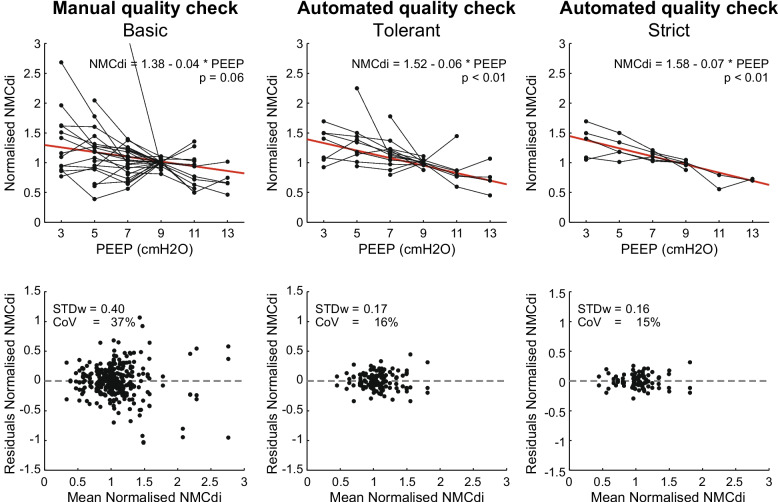
Effect of PEEP and quality criteria on NMCdi – Top: PEEP effect on NMCdi. Black lines show the median NMCdi per PEEP level within a measurement session (one patient, one day). In red the GEE-model is shown. Bottom: Residuals in NMCdi after subtraction of the mean NMCdi at that PEEP level for that patient at that day. NMCdi components PTPocc and ETPdi are unitless after normalisation to their median value at PEEP = 9 cmH_2_O. The residual plot after the manual quality check has two outliers outside the plotting range (mean; residual): (2.1; 1.5) and (1.7; 2.1)

**Fig. 4:**
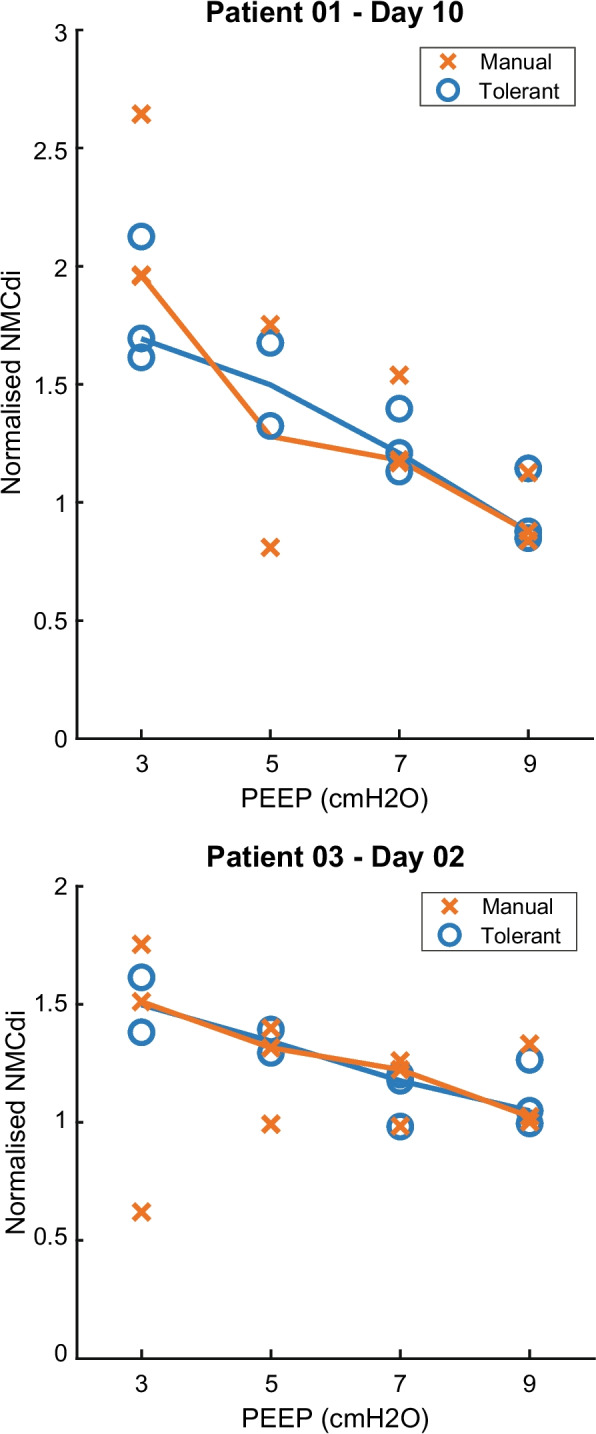
Effect of quality criteria on NMCdi on a patient level – Comparison of a basic manual quality check (manual) relative to an automated tolerant quality check (tolerant). NMCdi components PTPocc and ETPdi are unitless after normalisation to their median value at PEEP = 9 cmH_2_O

The CoV dropped considerably from 37% for basic manual selection to 16% for tolerant quality criteria and to 15% for strict criteria (Fig. 3). Within individual PEEP steps, the NMCdi variation decreased accordingly, and outliers were eliminated (Fig. 4).

### PEEP effect on NMCdi

When manually excluding only those sEAdi recordings that showed no respiratory activity, a non-significant (*p* = 0.06) effect of PEEP on normalised NMCdi was found, although a negative trend was visible (Fig. 3, manual basic). The improved parameter calculation and automated and objective quality assessment resulted in a significant trend (*p* < 0.01) when applying the tolerant quality criteria, which translates into a NMCdi decrease of 4.3 percentage point (pp) per cmH_2_O of PEEP relative to a PEEP of 3 cmH_2_O. This trend became even stronger, a reduction of 4.9 pp per cmH_2_O, when applying the strict quality criteria (Fig. 3).

## Discussion

This study shows that advanced analysis of both Pocc and sEAdi waveforms allows for automated identification of high-quality waveforms, reducing NMCdi variability to 15%. Waveforms were primarily excluded based on poor Pocc manoeuvre quality, and on low SNR or high AUB of the sEAdi waveforms. These results suggest the practical feasibility of adequate dataset selection from completely non-invasive EMGdi recordings in an intensive care environment to comprehensively assess the electromechanical activity of the respiratory muscle pump in critically ill patients.

### Defining reliable NMCdi

Measurements of NMCdi based on Pocc have been hampered by an unacceptably high variability, mainly caused by non-physiological waveforms. No practically feasible mathematical approach was described so far to identify and eliminate these waveforms and decrease variability of NMCdi [11]. The current study presents criteria that enable automated detection of sEAdi and Pocc waveforms that are credible from a physiological perspective as based on features of the overall curves as well as their temporal and mechanistic relation.

NMCdi calculation is based on the match between the mechanical output (Pocc) and its respective electrical activation (sEAdi). Waveforms showing abrupt or irregular releases of Pocc resulting in a mismatch with its sEAdi peak in terms of timing, duration or morphology were excluded.

To ascertain adequate ETPdi calculation from the sEAdi peaks through distinction from their background noise, waveforms with a low SNR or a high area under the baseline were excluded. The remaining sEAdi waveforms were expected to have a morphology as described in physiological literature: a gradual increase up to the maximal inspiratory activity, after which the sEAdi decreases gradually [12]. Waveforms were also excluded if they did not resemble a bell-shape, as mathematical equivalence of this physiological pattern. This bell-shape criterion detected highly irregular peaks, which could be ascribed to remaining ECG artifacts. Although, the bell-shape is a mathematical simplification of the physiological behaviour of the sEAdi waveform, this approach showed that variations in peak morphology were well tolerated, as for example slight tilting of the curve resulted in only small, subthreshold, ‘errors’.

The main reasons to exclude waveforms from further analysis were irregularities in Pocc, and a low SNR or high AUB in the sEAdi waveform. The latter two were highly prevalent in patients with an above median BMI. In these patients, the increased thickness of the subcutaneous skin layer may introduce an increased distance between the electrodes and the diaphragm, yielding lower surface potentials of diaphragm activity [13]. Although electrode positioning in this study was in accordance with general sEMG guidelines [14] and sEAdi studies [15], changing to a bilateral setup with larger inter-electrode distances and pick-up areas [6, 7], could yield higher sEAdi amplitudes and improve SNRs.

### Physiological effect of PEEP on NMCdi

The advanced signal analysis approach as described here, allowed to demonstrate a significant decrease in non-invasive NMCdi in response to an incremental PEEP trial in ICU patients studied, consistent with the decrease that has been described before in healthy subjects [16]. Importantly, the NMCdi in these healthy subjects was calculated from invasive transdiaphragmatic pressure (Pdi) and EAdi and thus exclusively represented the physiological effects of PEEP on the diaphragm. In the current study, non-invasive NMCdi was calculated through PTPocc, which is the summed pressure output of all respiratory muscles, and not confined to the diaphragm. By definition (Eq. 1), a decrease in NMCdi can result from either impaired pressure output generated by the diaphragm for a constant ETPdi, i.e. diminished neuromuscular efficiency, or from a higher relative contribution of the diaphragm to the generated PTPocc. The latter would require the diaphragm to be recruited to a greater extent than other respiratory muscles. However, diaphragm muscle fibres are found to shorten during an incremental PEEP trial [16, 17], bringing the diaphragm into a mechanically disadvantageous loading position. This implies that the diaphragm is not able to increase its relative recruitment, reflecting an impaired neuromuscular coupling of the diaphragm at higher PEEP levels.

### Bedside respiratory effort monitoring

Previous studies have shown the stability of NMCdi over different support levels [18]. A close correlation was found between invasive and non-invasive NMCdi [6, 7], indicating the clinical potential of NMCdi as a non-invasive alternative for respiratory effort monitoring as compared to invasive modalities based on Pes and EAdi [6, 18]. The current study reports the decrease of NMCdi with an incremental PEEP trial not only on the group level, but also on an individual patient level. This highlights the importance of appraising the physiological mechanisms affecting NMCdi, which should be corrected for when calculating respiratory effort from non-invasive NMCdi. Further improvement of signal quality to decrease the reported high waveform exclusion rate is important to develop sEMGdi as a widely applicable monitoring modality for bedside monitoring of respiratory effort in the individual critical care patient. Direct screening of Pocc manoeuvre quality and low SNR during signal acquisition will decrease exclusion rates during future analysis. In addition, more liberal cut-off values for application of the quality criteria will decrease exclusion rates but also increase variability, which can be compensated by averaging more repetitions [7, 11]. Alternatively, recent studies show that robustness of effort assessment can also be improved by integrating information from multiple data sources, such as ventilator data [19] and sEMG recordings of additional respiratory muscles [6].

### Limitations

The sample size of this study was relatively small, but is very comparable to other feasibility studies on sEAdi in the ICU [7, 9]. By excluding patients with a BMI > 30 kg/m^2^, the studied population not fully represents the general ICU population. Importantly, the included patients form a heterogeneous population with various admission reasons that are known to exhibit longer weaning durations, making these patients in particular need of continuous respiratory effort monitoring [20]. It should be noted that motor restlessness or discomfort can introduce significant crosstalk on sEMG leads, reduce SNR, and even counteract the imposed PEEP when, for example, the abdominal muscles are recruited (see Additional File 1) [16]. Patients experiencing discomfort often show higher respiratory drive [21], which could affect Pocc performance in terms of intensity and duration of inspiratory effort. The applied criteria in this study potentially caused non-random missing data in this specific population, although the mechanistic effect of PEEP on NMCdi is not expected to be fundamentally different in these patients.

## Conclusion

Advanced waveform analysis of both Pocc and sEAdi reduced NMCdi variability and thereby improved the robustness of sEMG based respiratory effort measurements by automated identification of high-quality occlusion manoeuvres. Poor Pocc manoeuvre quality and low sEAdi signal-to-noise ratio were the main contributors to high waveform exclusion rates. This automated approach allowed to demonstrate a significant decrease in NMCdi with increasing PEEP levels in mechanically ventilated ICU patients, as previously found in healthy subjects. These findings signify the importance of accounting for PEEP related diaphragm function when evaluating sEMG based respiratory effort to optimise ventilatory support in individual patients. This novel, non-invasive approach forms an important methodological foundation towards continuous and comprehensive bedside monitoring of respiratory effort, based on sEAdi, in ICU patients.

### Supplementary Information

**Additional file 1:** Signal processing.

Additional file 1: Signal analysis pipelines. Binders online available at: https://github.com/ReSurfEMG/binders.

**Additional file 3:** Pre-measurement patient characteristics.

## Data Availability

The datasets used and/or analysed during the current study are available upon reasonable request.

## Abbreviations

MV: Mechanical ventilation
ICU: Intensive care unit
Pmus: Respiratory muscle pressure
Pes: Oesophageal pressure
EAdi: Electrical activity of the diaphragm
NMCdi: Neuromechanical coupling of the diaphragm
Pocc: End-expiratory occlusion pressure
P0.1: Inspiratory occlusion pressure in the first 100 ms
sEMG: Surface electromyography
sEMGdi: SEMG of the diaphragm
sEAdi: Electrical activity of the diaphragm, envelope of sEMGdi signal
PEEP: Positive end-expiratory pressure
CCMO: Central Committee on Research Involving Human Subjects
FiO2: Fraction of inspired oxygen
SpO2: Oxygen saturation
BMI: Body mass index
RASS: Richmond Agitation and Sedation Scale
ECG: Electrocardiogram
Paw: Ventilator airway pressure
F: Ventilator flow
V: Ventilator volume
PTPocc: Pressure-time-product of Pocc
ETPdi: Electrical-time-product of sEAdi
AUB: Area under the baseline
SNR: Signal-to-noise ratio
Tdi: Median time between sEAdi peaks
T_HR_: Median time between QRS-complexes
IQR: Interquartile range
GEE: Generalised estimating equations
CoV: Coefficient of variation
PE: Pulmonary embolism
TBI: Traumatic brain injury
COPD: Chronic obstructive pulmonary disease
AAA: Abdominal aortic aneurysm
pp: Percentage point
Pdi: Diaphragmatic pressure
