# Supplementary material for: Advanced waveform analysis of diaphragm surface EMG allows for continuous non-invasive assessment of respiratory effort in critically ill patients at different PEEP levels
Source: Crit Care. 2024 Jun 9;28:195. doi: 10.1186/s13054-024-04978-0 (PMC11162564; doi:10.1186/s13054-024-04978-0)
Supplement: Supplementary file 1 — Additional file 1: Signal processing. [file 13054_2024_4978_MOESM1_ESM.docx]

Additional file 1 – Signal processing

# Processing of ventilator signals

### PEEP setting and moving baseline

The PEEP-level was automatically detected as the median airway pressure (Paw) at end-expiration, defined as the local minimum in ventilator volume (V). PEEP was rounded down to the nearest integer. The moving baseline of airway pressure (Paw) was taken as the reference line relative to which occlusion pressure on-and offset were detected (Paw < Pref). The moving baseline over Paw was calculated as the 33^rd^-percentile over a centralised window of 7.5 s. A step-size of 0.2 s, which translates to 20 samples, was taken to speed up the moving baseline calculation, as the baseline showed little variation over the course of several samples.

### Pocc peak detection and PTPocc calculation

Occlusion pressure peaks were automatically detected in Paw by thresholding negative pressure deflections relative to the detected PEEP-level. The detection threshold was set at a minimal peak height and prominence of 30% of the maximal (negative) amplitude. This threshold percentage was decreased in case there was a considerable difference between Pocc amplitudes. If the threshold was also exceeded during the trigger phase of supported breaths, the threshold was increased. Pocc on- and offset were detected as the Paw moving baseline crossings directly before and after the detected peak pressure. PTPocc was calculated as the area between Paw and the moving baseline between the detected on- and offset. The supplemental area above the baseline – above as Paw deflects negatively during an occlusion – is calculated as the area between the moving baseline and the maximum value of the moving baseline in a window 5s before and after the detected Pocc peak.

### Pocc quality criteria

Irregular cessation of the inspiratory effort was evaluated if the lower decile of the Paw upslope, i.e. the slope between the occlusion peak and the offset, was below zero. This would indicate a significant proportion of samples with a negative slope, whereas the upslope should be strictly increasing.

Abrupt cessation of the inspiratory effort was evaluated by the upslope of Paw normalised relative to the calculated PTPocc. If the upper decile of the upslope, i.e. highest slope between the occlusion peak and the offset, was larger than 80% of $\sqrt{PTPocc}$, the peak was excluded. Impermissible peaks displayed a high slope during the majority of samples, while samples with lower slopes were nearly absent. As a result, the upper decile distinguished abrupt cessations of the occlusion manoeuvre from permissible manoeuvres. Normalisation relative to $\sqrt{PTPocc}$ was needed to allow for high slopes in case of a strongly negative Pocc.

# Processing of sEMG signals

### QRS complex detection

QRS-complexes were detected in the ECG lead. This ECG signal was band-pass filtered from 1 to 500 Hz, thereby removing baseline wander, but leaving the QRS morphology intact. The resultant signal was filtered with a 10 sample (~0.005 s) centralised RMS filter to allow for QRS-peak detection, independent of the electrical heart axis. The peak detection threshold was initialised at 30% of maximal RMS amplitude, and adapted per case to discriminate between QRS- and other ECG-segments. Minimum peak distance was set at 0.33 s. The detected QRS-complexes were gated out in 100 ms (205 samples) windows. The gates were filled with the running RMS over twice the gating window, centralised around the respective sample, excluding the samples within the gated window itself. For example, the sample halfway the gate is set to the RMS over half the window before and half the window after the gate. Over the resulting signal, the envelope (sEAdi) was calculated using a moving 200 ms RMS filter.

### Peak detection

Respiratory activity in the sEAdi was automatically detected according to a height and prominence threshold, proportional to the maximal sEAdi amplitude in the recording. The threshold was manually set in order to detect the sEAdi peaks associated with respiratory efforts, i.e. occlusion pressures and supported breaths. The minimal peak duration was set at 0.5 s. On- and offset were detected as the sEAdi moving baseline crossings directly before and after the detected peak pressure.

### Improved moving baseline

At first, the moving baseline over the sEAdi was calculated in accordance with [1] using a 33^rd^ percentile over a 5 s window. This resulted in ill-behaved on- and offset detection in some traces as illustrated in Figure 1B of the main article. Two main factors were identified to contribute to this behaviour, and the moving baseline was adapted accordingly.

- Whereas respiratory sEAdi peaks are characterised by a rapid up- and downslope, some peaks were preceded by a gradual increase in sEAdi which already crossed the moving baseline before commencement of the pneumatic inspiratory effort, which was mainly due to baseline wander. As the moving baseline was calculated over the sEAdi and not over its rate of change, it often did not discriminate between the slow behaviour of the wandering baseline and the rapid up- and downslope of the respiratory activity. To introduce a rate of change component, an augmented signal is proposed to calculate the baseline, consisting of the sEMG signal and its smoothed first derivative:

$$\mathrm{sEA}_{\mathrm{aug}}\left( k \right)= sEAdi\left( k \right)+\frac{1}{W_{\mathrm{MA}}}\sum_{i=-\frac{W_{\mathrm{MA}}}{2}}^{\frac{W_{\mathrm{MA}}}{2}} \left| \mathrm{sEAdi}\left( i+k \right)-sEAdi\left( i+k+1 \right) \right|$$

With moving average window $W_{\mathrm{MA}}=\frac{1}{2}*fs$

The augmented baseline is then calculated as 120% of the 25^th^ percentile over a 7.5 s window:

$$\mathrm{baseline}_{\mathrm{aug}}\left( k \right)=1.2*med\left( \mathrm{sEA}_{\mathrm{aug}}\left( i \right) \right), i \epsilon k\pm\frac{W_{\mathrm{MB}}}{2}$$

With moving baseline window $W_{\mathrm{MB}}=7.5*fs$

The moving average window ($W_{MA}$) of 0.5 s, moving baseline window ($W_{MB}$) of 7.5 s and amplification factor 1.2 were empirically found to result into robust on- and offset detection.

- Moreover, local noisiness of the sEMG signal introduced significant uncertainty in the detected on- and offset. In such cases, a higher threshold is needed for robust detection. Therefore, the augmented baseline was amplified by the coefficient of variation of the baseline times the median sEMG peak height normalised to the baseline level (sEMGpeak / baseline mean):

$\mathrm{baseline}_{aug, final}\left( k \right)=\mathrm{baseline}_{\mathrm{aug}}\left( k \right)*(1+CoV\left( k \right)*AmpNorm\left( k \right))$

Where the coefficient of variation is defined as:

$\mathrm{CoV}\left( k \right)=\frac{\mathrm{var}\left( \mathrm{baseline}_{\mathrm{aug}}\left( i \right) \right)}{mean(\mathrm{baseline}_{\mathrm{aug}}\left( i \right)}, i \epsilon k\pm\frac{W_{\mathrm{MB}}}{2}\mathrm{fs}$,

And the amplitude normalisation factor:

$$\mathrm{AmpNorm}\left( k \right)=\frac{\mathrm{med}\left( \mathrm{sEAdi}\left( p \right) \right)}{mean(\mathrm{baseline}_{\mathrm{aug}}\left( i \right)},$$

$$With i \epsilon k\pm\frac{W_{\mathrm{MB}}}{2}fs and p:=sEMG peak indices$$

Normalisation to peak height was required to keep the baseline in the same range as the sEMG signal. sEMG on- and offsets were consequently defined as the samples where $\mathrm{sEAdi}_{\mathrm{aug}}$ crossed $\mathrm{baseline}_{aug, final}$.

### ETP calculation

ETPdi was calculated as the area between sEAdi and the moving baseline, between the detected on- and offset, plus the area under the baseline (AUB). The AUB was defined as the area between the moving baseline and the sEAdi minimum in the window from 5 s before the onset to 5 s after the offset.

### Quality criteria

#### Signal-to-noise ratio (SNR)

The SNR of sEAdi was approximated by the peak height divided by the median of the moving baseline in a 1 s window around the peak:

$$\mathrm{SNR}\left( k \right)=\frac{\mathrm{sEAdi}\left( k \right)}{\mathrm{med}\left( \mathrm{baseline}_{aug, final}\left( i \right) \right)}, i \epsilon k\pm\frac{1}{2}\mathrm{fs}$$

*Interpeak distance*

The interpeak distance for the sEAdi (Tdi) and the heartrate (T_HR_) were defined as the median interpeak interval over all detected respiratory efforts and QRS-complexes, respectively. For Tdi all detected respiratory efforts as described in section 2.2 were used, and for T_HR_ the detected QRS-complexes as described in section 2.1.

#### Area under the baseline (AUB)

The AUB is calculated as defined in section 2.4. The relative AUB for the quality criterion was calculated as the AUB as fraction of the total ETPdi, i.e. AUB and area between sEAdi and the moving baseline.

Bell-morphology

A bell-curve was fit to the sEAdi curve between on- and offset, and the summed-absolute-error (SAE) was calculated according to:

$$SAE= \sum_{k=onset}^{\mathrm{offset}} \left| \mathrm{sEAdi}\left( k \right)-\mathrm{bell}\left( k \right) \right|$$

With $\mathrm{bell}\left( k \right)=a{*e}^{-\frac{\left( k-b \right)^{2}}{c^{2}}}, with a \epsilon\left[ 0, 60 \right], b \epsilon\left[ -\frac{\mathrm{fs}}{2} ,\frac{\mathrm{fs}}{2} \right] and c \epsilon\left[ 0, 0.5 \right]$

This required the bell-shape to be positive ($a \epsilon\left[ 0, 60 \right])$, and have its maximum within 0.5 s of the detected peak ($b \epsilon\left[ -\frac{fs}{2} ,\frac{fs}{2} \right])$. $c \epsilon\left[ 0, 0.5 \right]$ was empirically found to result into proper fits. The Bell-error was defined as:

$$Bell error=\frac{\mathrm{SAE}}{\mathrm{ETPdi}}$$

# Performance automated quality assessment

## Manual versus automated

The number of in- and excluded manoeuvres by the manual and automated quality assessment in the confusion matrix shown below. This table shows that nearly all manoeuvres included in the automated checks were also included in the manual checks. The automated checks are generally stricter, mainly due to a low signal-to-noise ratio and a high area under the baseline.

|  | | Automated | | | |
| --- | --- | --- | --- | --- | --- |
|  |  | Tolerant | | Strict | |
|  |  | Excluded | Included | Excluded | Included |
| Manual | Excluded | 277 | 4 | 281 | 1 |
|  | Included | 139* | 173 | 195** | 116 |
| * Specification of tolerant automated exclusion reasons:  Pocc: 43, SNRdi: 22, Tdi: 1, AUB: 65, Bell morphology: 8  ** Specification of strict automated exclusion reasons:  Pocc: 43, SNRdi: 77, Tdi: 1, AUB: 68, Bell morphology: 6 | | | | | |

## Crosstalk detection

Considering that contamination by other muscle activity is common in respiratory surface EMG [2], the behaviour of the quality assessment methodology was evaluated in some exemplary cases. Disturbance of other muscles can mainly be attributed to the abdominal muscles. In general, abdominal muscle activation is to be expected due to (forced) expiration, which is out of phase with the inspiratory activity of the diaphragm, or due to motor restlessness, which occurs across the respiratory cycle.

In several cases we observed patients actively exhaling, using their abdominal muscles. An example of such recording is provided in the figure below. The ventilator pressure and volume are shown in the upper two plots of the graph, with the corresponding sEAdi signal in plot 3. Cyclic sEAdi activity can be observed both during the expiratory phases (red shade), as well as lower activity during the inspiratory phase (green shade). As a result of both inspiratory and expiratory activity in the sEAdi signal, the mean signal power increases, pushing the moving baseline upwards. The area under the baseline of the inspiratory diaphragm activity increases along with the moving baseline, making it more likely for these manoeuvres to be excluded because of a high area under the baseline. Consequently, only the inspiratory sEAdi that are sufficiently high in amplitude were acceptable for inclusion.


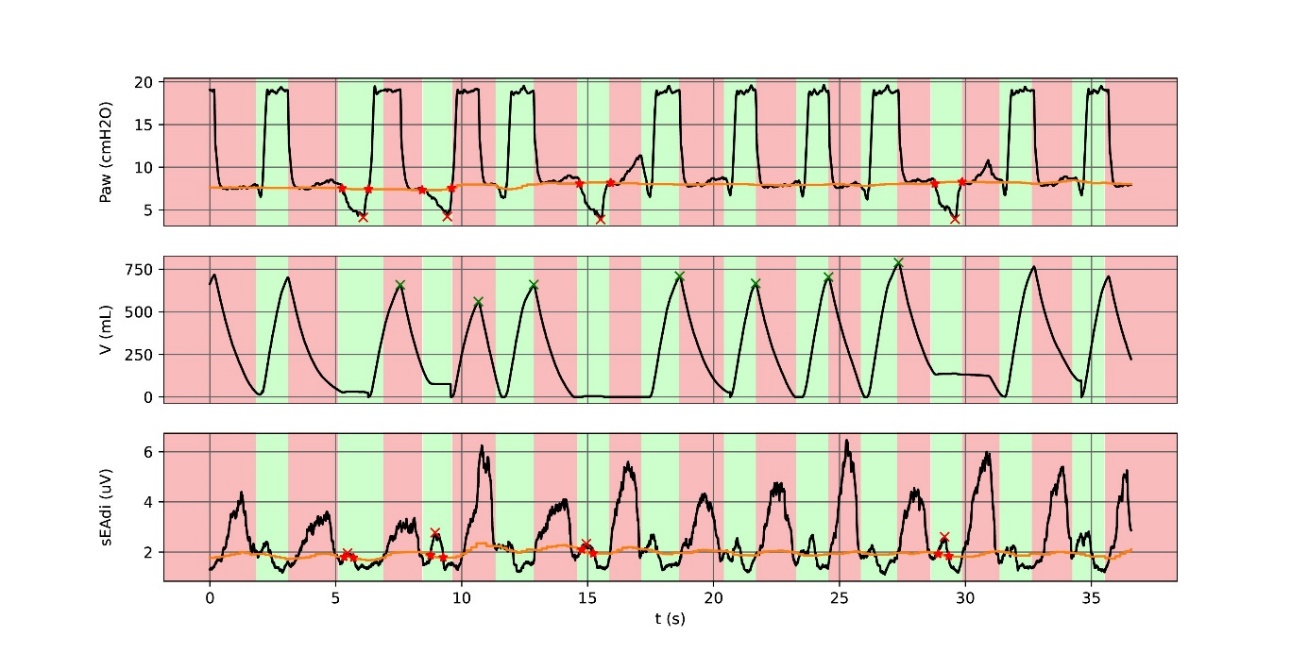


Figure 1 - Timing of activity during expiratory (red) and inspiratory (green) phases. Red markers indicate the detected occlusion pressures.

Motor restlessness, being the other main contributor to (abdominal) muscle activation, is generally present during one or more respiratory cycles. Although, restlessness during the measurements was always a reason to wait until calmness resumed and the end-expiratory occlusion manoeuvres could be performed; some datasets reflect such scenarios of contamination in between occlusion manoeuvres. This crosstalk increased baseline activity resulting in a reduced the signal-to-noise ratio. The figure below shows that manoeuvres in windows with high abdominal activity (red shade) which will be most likely excluded because of a low SNR and high area under the baseline.


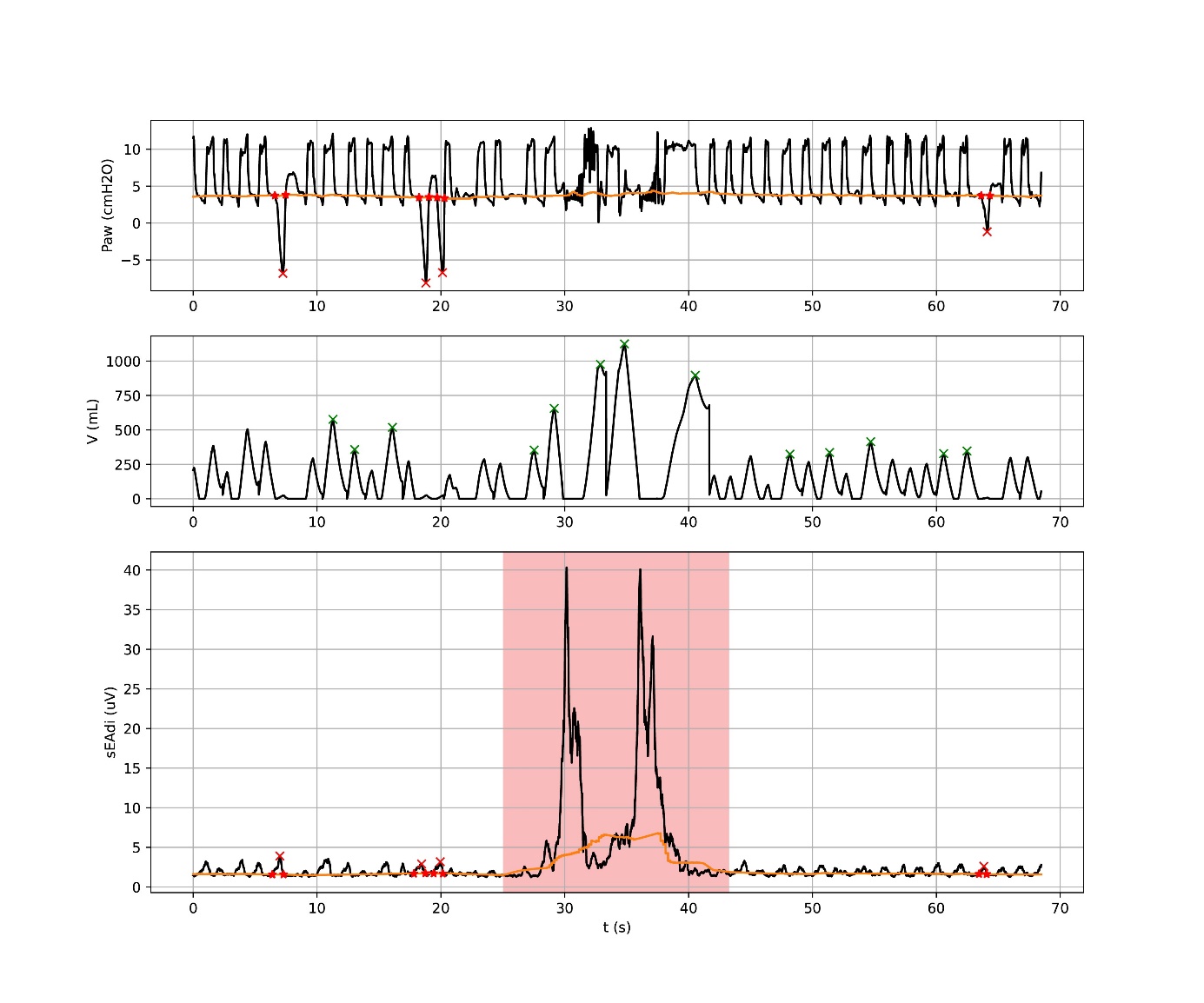


Figure 2- Motor restlessness results in high level activity and a high baseline throughout the respiratory cycles (red shading). Red markers indicate the detected occlusion manoeuvres.

# References

[1] J. Graßhoff *et al.*, “Surface EMG-based quantification of inspiratory effort: a quantitative comparison with Pes,” *Crit Care*, vol. 25, no. 1, pp. 1–12, Dec. 2021, doi: 10.1186/s13054-021-03833-w.

[2] A. H. Jonkman *et al.*, “Analysis and applications of respiratory surface EMG: report of a round table meeting,” *Critical Care 2023 28:1*, vol. 28, no. 1, pp. 1–17, Jan. 2024, doi: 10.1186/S13054-023-04779-X.
