# Supplementary material for: Advanced waveform analysis of diaphragm surface EMG allows for continuous non-invasive assessment of respiratory effort in critically ill patients at different PEEP levels
Source: Crit Care. 2024 Jun 9;28:195. doi: 10.1186/s13054-024-04978-0 (PMC11162564; doi:10.1186/s13054-024-04978-0)

Table 1 - Included and excluded measurements per patient

|  |  | **Pre measurement values** | | | | | **Included** | | | | |
| --- | --- | --- | --- | --- | --- | --- | --- | --- | --- | --- | --- |
| **Subject** | **Day** | **SpO2** | **FiO2** | **RASS** | **PEEP** | **PS** | **Manual** | **Tolerant** | | **Strict** | |
| 1 | 0 | 94 | 30 | -1 | 6 | 18 | 1 | 1 | | 1 | |
| 1 | 3 | 98 | 35 | 0 | 5 | 5 | 1 | 0 | | 0 | |
| 1 | 5 | 99 | 35 | 0 | 5 | 8 | 1 | 0 | | 0 | |
| 1 | 10 | 99 | 35 | 0 | 5 | 5 | 1 | 1 | | 1 | |
| 3 | 0 | 94 | 45 | -2 | 5 | 10 | 1 | 1 | | 1 | |
| 3 | 2 | 96 | 50 | -4 | 5 | 12 | 1 | 1 | | 1 | |
| 3 | 5 | 92 | 50 | 0 | 6 | 14 | 1 | 0 | | 0 | |
| 3 | 7 | 95 | 45 | 0 | 8 | 10 | 1 | 1 | | 0 | |
| 4 | 0 | 97 | 40 | -4 | 8 | 12 | 1 | 0 | | 0 | |
| 4 | 5 | 96 | 40 | -4 | 8 | 6 | 1 | 1 | | 0 | |
| 4 | 7 | 95 | 40 | 0 | 8 | 6 | 1 | 0 | | 0 | |
| 4 | 10 | 98 | 40 | 0 | 6 | 8 | 1 | 1 | | 1 | |
| 6 | 0 | 93 | 45 | -4 | 10 | 8 | 1 | 1 | | 0 | |
| 7 | 3 | 97 | 35 | -1 | 6 | 10 | 1 | 0 | | 0 | |
| 7 | 7 | 99 | 35 | -1 | 5 | 5 | 1 | 1 | | 0 | |
| 9 | 0 | 100 | 55 | -4 | 8 | 6 | 1 | 0 | | 0 | |
| 9 | 0 | 100 | 55 | -4 | 8 | 6 | 1 | 0 | | 0 | |
| 10 | 4 | 95 | 45 | -4 | 8 | 4 | 1 | 0 | | 0 | |
| 11 | 0 | 90 | 40 | 0 | 10 | 4 | 1 | 1 | | 0 | |
| 12 | 0 | 99 | 35 | -4 | 10 | 8 | 1 | 0 | | 0 | |
| 12 | 2 | 98 | 30 | -2 | 8 | 6 | 1 | 0 | | 0 | |
| 13 | 0 | 99 | 40 | -1 | 10 | 12 | 1 | 1 | | 1 | |
| 14 | 0 | 99 | 30 | 0 | 5 | 6 | 1 | 0 | | 0 | |
| 16 | 0 | 94 | 45 | -2 | 5 | 6 | 1 | 0 | | 0 | |
| 17 | 5 | 99 | 40 | -2 | 10 | 8 | 1 | 1 | | 1 | |
| 17 | 10 | 100 | 35 | -1 | 6 | 8 | 1 | 1 | | 0 | |
| 2 | 0 | 100 | 45 | -2 | 10 | 10 | 0 | 0 | | 0 | |
| 2 | 3 | 100 | 40 | 0 | 5 | 6 | 0 | 0 | | 0 | |
| 5 | 0 | 96 | 40 | 0 | 10 | 8 | 0 | 0 | | 0 | |
| 6 | 9 | 96 | 35 | -4 | 10 | 6 | 0 | 0 | | 0 | |
| 7 | 0 | 99 | 35 | -3 | 6 | 10 | 0 | 0 | | 0 | |
| 8 | 0 | 96 | 30 | -3 | 8 | 10 | 0 | 0 | | 0 | |
| 10 | 0 | 94 | 50 | -5 | 10 | 12 | 0 | 0 | | 0 | |
| 12 | 5 | 99 | 30 | 0 | 8 | 6 | 0 | 0 | | 0 | |
| 15 | 0 | 95 | 45 | 0 | 10 | 12 | 0 | 0 | | 0 | |
| 15 | 3 | 95 | 50 | -3 | 10 | 10 | 0 | 0 | | 0 | |
| 15 | 7 | 94 | 30 | 0 | 8 | 6 | 0 | 0 | | 0 | |
| 17 | 0 | 95 | 40 | -4 | 10 | 6 | 0 | 0 | | 0 | |
| 17 | 3 | 94 | 50 | -4 | 10 | 8 | 0 | 0 | | 0 | |
| 17 | 7 | 100 | 35 | -2 | 6 | 10 | 0 | 0 | | 0 | |
| 17 | 12 | 100 | 40 | 0 | 6 | 6 | 0 | 0 | | 0 | |
| 17 | 14 | 99 | 35 | 0 | 6 | 6 | 0 | 0 | | 0 | |
|  |  |  |  |  | N patients: | | 13 | | 8 | | 5 |
|  |  |  |  |  |  |  | 76% | | 47% | | 29% |
|  |  |  |  |  | N datasets: | | 26 | | 13 | | 7 |
|  |  |  |  |  |  |  | 62% | | 31% | | 17% |

Abbreviations: SpO2 Oxygen saturation, FiO2 fraction inspired oxygen, RASS Richmond Agitation and Sedation Scale, PEEP positive end-expiratory pressure, PS pressure support

## Mechanical ventilation duration

The duration of invasive mechanical ventilation (MV) varied considerably between patients at the time of inclusion (see Table 3 of the main article). To evaluate whether MV duration was associated with higher chances of manoeuvre exclusion, the sEMG quality aspects of all manoeuvres (signal-to-noise ratio, area under the baseline, bell morphology error and time between sEAdi peaks) were plotted versus the duration of invasive mechanical ventilation (MV) at the time of the measurement. The results are shown in the figure below. No obvious patterns of the quality aspects are yielded regarding the duration of MV.


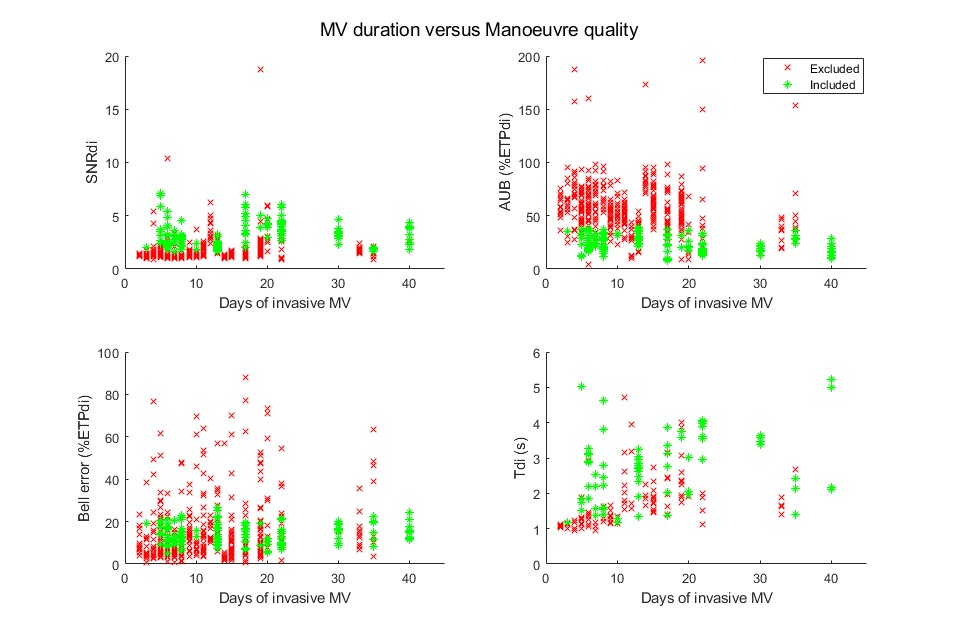

Supplement: Supplementary file 3 — Additional file 3: Pre-measurement patient characteristics. [file 13054_2024_4978_MOESM3_ESM.docx]
